# Evaluation of an On-Demand Mental Health System for Depression Symptoms: Retrospective Observational Study

**DOI:** 10.2196/17902

**Published:** 2020-06-18

**Authors:** Sarah Kunkle, Manny Yip, Watson Ξ, Justin Hunt

**Affiliations:** 1 Ginger San Francisco, CA United States

**Keywords:** mental health, depression, digital health, therapy, coaching, behavioral health, virtual care

## Abstract

**Background:**

Depression is an extremely prevalent issue in the United States, with an estimated 7% of adults experiencing at least one major depressive episode in 2017. Although psychotherapy and medication management are effective treatments for depression, significant barriers in accessing care persist. Virtual care can potentially address some of these obstacles.

**Objective:**

We conducted a preliminary investigation of utilization characteristics and effectiveness of an on-demand health system for reducing depression symptoms.

**Methods:**

Data were analyzed from 1662 users of an on-demand mental health system that includes behavioral health coaching, clinical services (therapy and psychiatry), and self-guided content and assessments primarily via a mobile app platform. Measures included engagement characterized by mobile app data, member satisfaction scores collected via in-app surveys, and depression symptoms via the Patient Health Questionnaire-2 (PHQ-2) at baseline and 8-12 week follow-up. Descriptive statistics are reported for measures, and pre/post-PHQ-2 data were analyzed using the McNemar test. A chi-square test was used to test the association between the proportion of individuals with an improvement in PHQ-2 result and care modality (coaching, therapy, and psychiatry, or hybrid).

**Results:**

During the study period, 65.5% of individuals (1089/1662) engaged only in coaching services, 27.6% of individuals (459/1662) were engaged in both coaching and clinical services, 3.3% of individuals (54/1662) engaged only in clinical services, and 3.7% of individuals (61/1662) only used the app. Of the 1662 individuals who completed the PHQ-2 survey, 772 (46.5%) were considered a positive screen at intake, and 890 (53.6%) were considered a negative screen at intake. At follow-up, 477 (28.7%) of individuals screened positive, and 1185 (71.3%) screened negative. A McNemar test showed that there was a statistically significant decrease in the proportion of users experiencing depressed mood and anhedonia more than half the time at follow-up (*P*<.001). A chi-square test showed there was no significant association between care modality and the proportion of individuals with an improvement in PHQ-2 score.

**Conclusions:**

This study provides preliminary insights into which aspects of an on-demand mental health system members are utilizing and levels of engagement and satisfaction over an 8-12 week window. Additionally, there is some signal that this system may be useful for reducing depression symptoms in users over this period. Additional research is required, given the study limitations, and future research directions are discussed.

## Introduction

Depression is one of the most prevalent and impactful health conditions in the United States, according to multiple data sources. Conservative estimates from national surveys show that 7%-8% of American adults are affected by major depression annually [1,2]. A national health index of more than 41 million people, compiled by one of the largest health insurance providers, ranked depression second only to hypertension in its impact on American longevity and quality of life [3]. In addition to the direct symptoms caused by depression, the majority of adults with depression report at least some difficulty with work, home, or social activities due to their symptoms [2]. Thus, the health burden of depression translates into a significant social and economic burden [4].

### Existing Approaches and Barriers

Although there are effective treatments (including pharmacological and psychotherapy interventions) for depression and other mental health conditions, the current US healthcare system does not adequately address the many social, medical, and economic barriers. Access to formal behavioral health treatment remains a challenge because of a complex collection of barriers, including perceived public or social stigma, inadequate behavioral health workforce in certain geographic regions, and poor insurance coverage and related financial costs [5].

The mental health workforce shortage is more acute for some geographic regions; nearly 40 percent of Americans live in areas designated by the federal government as having a shortage of mental health professionals [6,7]. Access challenges include general barriers such as high cost, time, and transportation, but also specific obstacles like stigma and treatment preference [8]. Having access to a preferred choice of treatment improves treatment initiation, adherence, and outcomes [9,10].

#### Collaborative Care Model

The primary care-based collaborative care model augments the model of behavioral health care being provided in primary care and replaces it with a three-pronged team, including the primary care physician, a care manager, and a psychiatric consultant. This model helps to spread the expertise of the psychiatric consultant across a population of primary care patients through the actions of the care manager and the prescribing of the primary care provider (PCP); therefore, a larger population of primary care patients can receive evidence-based mental health care [11].

While the collaborative care model has a strong evidence base and is beginning to receive recognition from large payors such as Centers for Medicare & Medicaid Services (CMS), challenges remain in the real-world implementation of the model. In primary care settings, it remains difficult for overburdened PCPs to prescribe psychotropic medications in a 10-15 minute encounter that can often be focused on many chronic health conditions. It can also be challenging for a PCP to digest the recommendations of the care manager or psychiatric consultant team [12].

Beyond the challenges of limited time and competing priorities within a brief appointment, the collaborative care model only addresses those patients who present to primary care with behavioral health symptoms that rise to the level of a diagnosed DSM-V mental illness, such as Major Depression or Generalized Anxiety Disorder, or the symptoms are severe enough to trigger a full PHQ-9 or GAD-7. There is another “hidden” population of individuals suffering from upstream subclinical stress and behavioral health symptoms who do not present to a PCP because they do not have a comorbid medical problem, or they may not have easy geographic or financial access to a regular PCP. In fact, according to a 2018 Kaiser poll, many Millenials report not having and not even desiring a regular PCP [13].

#### Telehealth and Digital Interventions

The US healthcare system has attempted to close this chasm of unmet behavioral health needs and concurrent poor quality of care through a variety of different mechanisms, including telemedicine and other digital solutions. Telehealth has become the standard to address geographic barriers to care in rural regions over nearly three decades in systems such as the Department of Veteran Affairs, where evidence-based psychotherapy and medication management can be delivered at an equivalent level of quality as in-person care [14]. A 2016 systematic review showed that telephone-based interventions had promised in reducing symptoms of depression and anxiety [15]. Unfortunately, most telemental health models primarily address the geographic maldistribution of providers as opposed to the sheer lack of full-time providers across the country.

Smartphone-based treatments have also shown promise in managing depression according to a 2017 meta-analysis [16]. Given the heterogeneity of these interventions, many studies highlight the need to establish which aspects of these technologies are most active for a given population. Thus, characterizing the features, engagement, and users of specific products can help enhance our understanding of how these new technologies and systems work for different populations.

In the broader digital health landscape, there have been calls for companies to produce and publish evidence on outcomes, including engagement and clinical outcomes [17]. There are multiple dimensions for evaluating these types of products, and many organizations, including the US FDA, the UK NHS, the APA, have proposed frameworks to guide informed decision making and evaluation. Common categories of evaluation include privacy and security, evidence base, ease of use, and data integration [18].

This exploratory study aimed to investigate the initial effectiveness of a novel on-demand system by describing utilization and satisfaction measures in addition to evaluating changes in self-reported depression symptoms. Previous research has proposed some guidelines for conducting studies in real-world settings in contrast to highly controlled efficacy studies, including observing utilization, satisfaction, and outcomes in actual practice [19]. This is consistent with principles of implementation research, which seeks to understand intervention in real-world conditions, rather than trying to control for conditions or to remove their influence as causal effects [20]. Studies of other digital mental health technologies have used this approach to investigate initial effectiveness in addition to guiding future research directions [21,22].

## Methods

### Participants

Individuals in this study are employees or health plan members who have access to the Ginger system as part of their employer or health plan benefits. The Ginger system includes the following exclusionary criteria where telehealth is likely not appropriate:

1. Active suicidal ideation
2. Active high-risk self-harm behavior
3. Two or more hospitalizations within the past 6 months for psychiatric reasons
4. Certain symptoms of psychosis that are poorly managed (eg, the member is not med compliant, or symptoms are unresponsive to treatment), likely incompatible with telehealth
5. A primary diagnosis of a substance use disorder, or moderate to severe substance abuse issues, due to the high complexity, severity, and risk frequently associated with such members, as well as the need for specialized care
6. Active eating disorders with symptoms considered to be high risk
7. Ongoing grave disability, including bipolar patients with active mania/hypomania or mixed episodes who are unmedicated or who have poor compliance with medication regimen over time
8. Two or more medical hospitalizations in the last month due to the high likelihood that the individual has a poorly controlled medical condition that requires close monitoring

For this study, we included Ginger users aged 18 or older who downloaded the app between January 1, 2018, and December 31, 2019. This period was chosen as it reflects the approximate timing of when Ginger began to provide care to members via its employer business. Based on these criteria, 24,682 individuals were eligible for the study, 10,942 users (44.33%) completed the intake survey, and 1662 users (6.73%) completed a 12-week follow-up survey and were included in our analysis. Survey response rates are a challenge in both online studies and behavioral health practice; research has shown that the majority of participants in remote studies discontinue participation within the first week of a study and that online survey rates tend to be much lower than in-person [23,24]. The survey response rates here are lower than what is reported in the literature, which is expected given the observational nature of this study and the design of the survey system in a digital product, where users are not required to complete surveys to receive care.

### Procedures

#### The Ginger System

**Figure 1:**
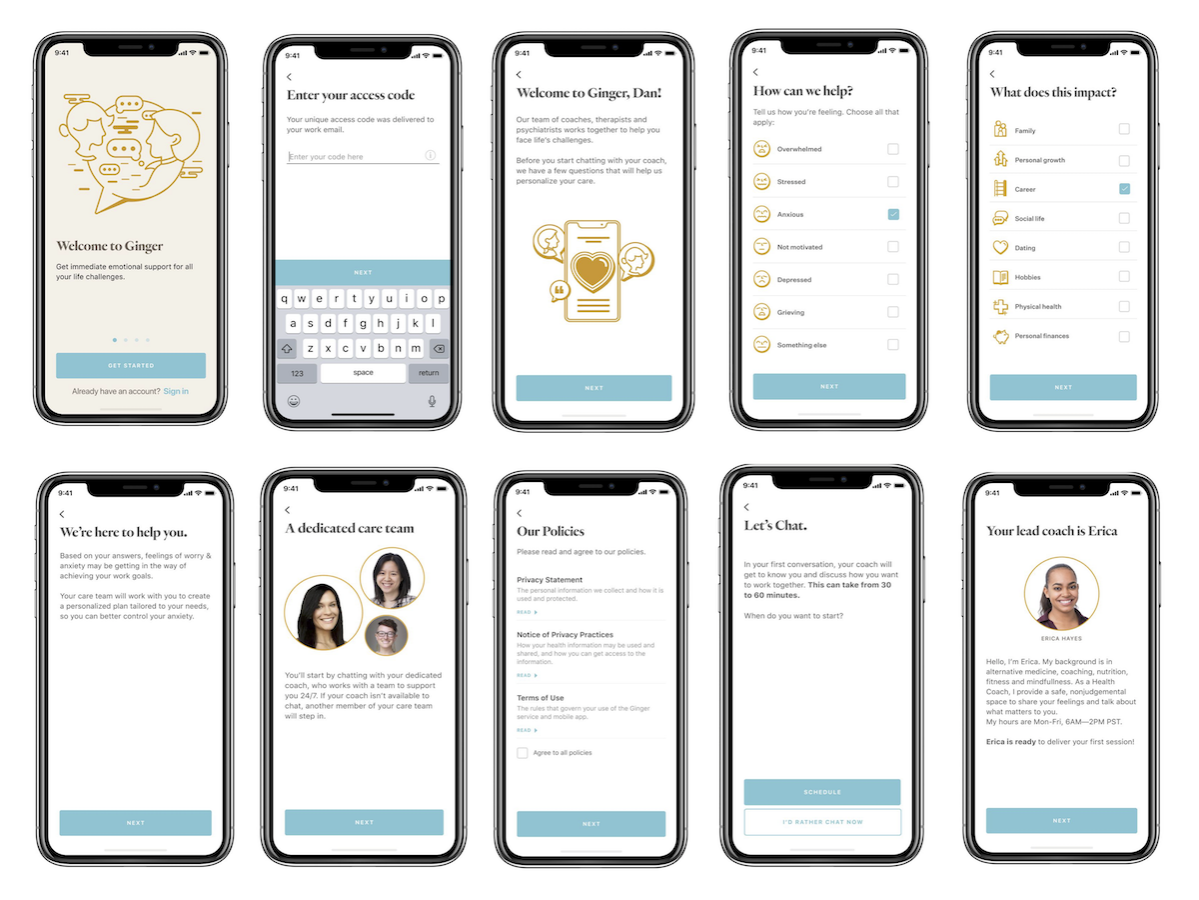
Ginger mobile app onboarding screens.

**Figure 2:**
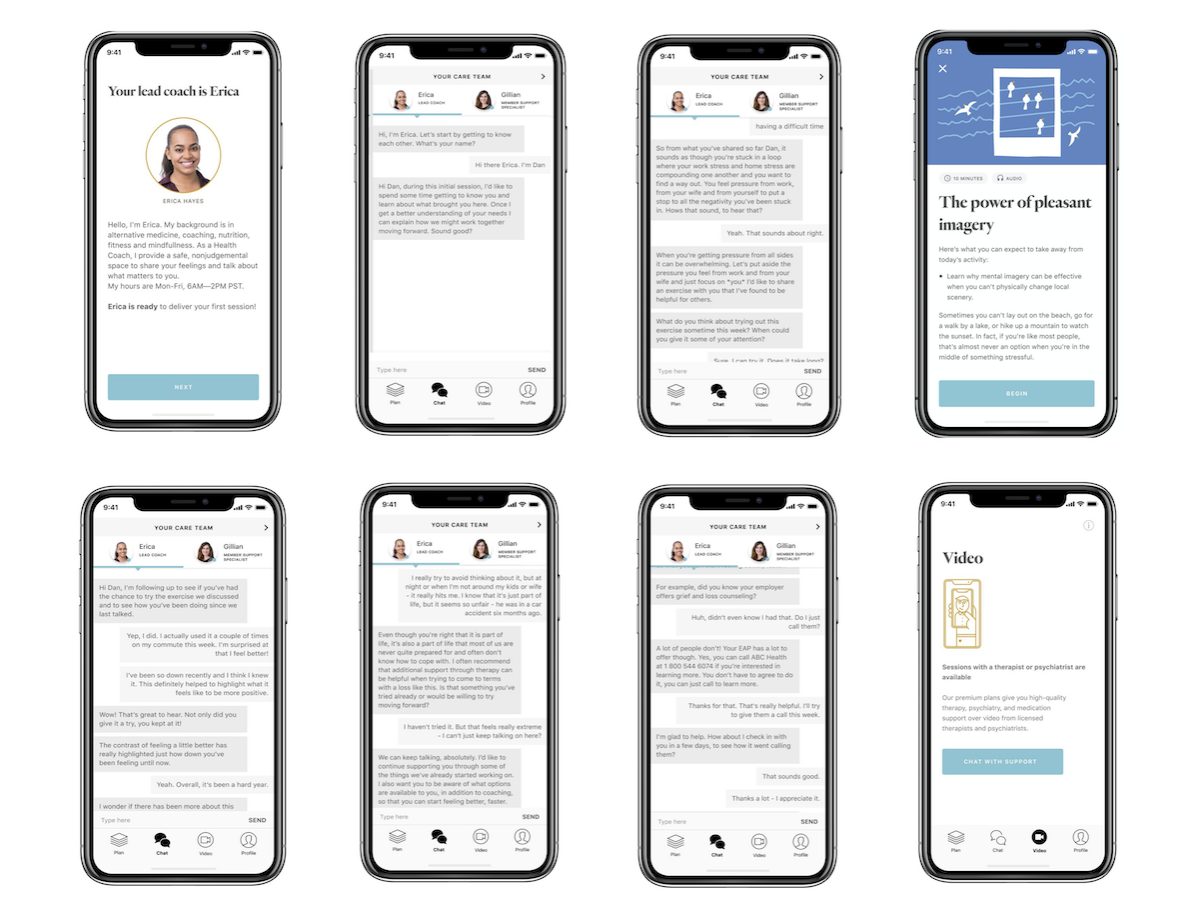
Ginger coach chat screens.

Ginger provides members with behavioral health coaching, therapy, and psychiatry, along with self-guided content and assessments primarily via a mobile app platform. Members generally have access to Ginger as part of their employee or health plan benefits. After downloading the mobile app, users can start chatting with a behavioral health coach within minutes (Figures 1 and 2). Ginger coaches are full-time employees who typically have an advanced degree in a field related to mental health, and an accredited Coach Certification [25,26]. While many users solely engage with coaches, some will request or require escalation to psychotherapy services. Examples of situations that require escalation include individuals with chronic mental illness and severe trauma, the potential of harm to self or others, and significant mental instability (ie, hallucinations, delusions, and extreme mood swings). When members are escalated to therapy or psychiatry, they may continue working with a coach on an on-demand basis, provided that they also agree to seek additional care concurrently; coaching can continue supporting them in addressing day to day goals and challenges, as well as acting as an adjunct to the care plan put in place by their therapist or psychiatrist.

### Data Collection

The Ginger system incorporates regular check-ins and feedback to understand better what solutions work for individual members, consistent with principles of measurement-based care (MBC). Although MBC has been demonstrated to enhance care, studies have shown that less than 20 percent of behavioral health practitioners integrate it into their practice [27]. Ginger assesses depression via the Patient Health Questionnaire (PHQ), delivered via the Ginger mobile app. The PHQ is one of the most validated assessments in mental health and is commonly used by clinicians in screening for and diagnosing depression in addition to monitoring treatment response [28]. When users onboard with the Ginger app (Figure 1), they are prompted first to answer the PHQ-2 questionnaire. Individuals that provide answers with a score of 2 or above for either question are considered a “positive depression screen” and then asked to complete the full PHQ-9.

**Figure 3:**
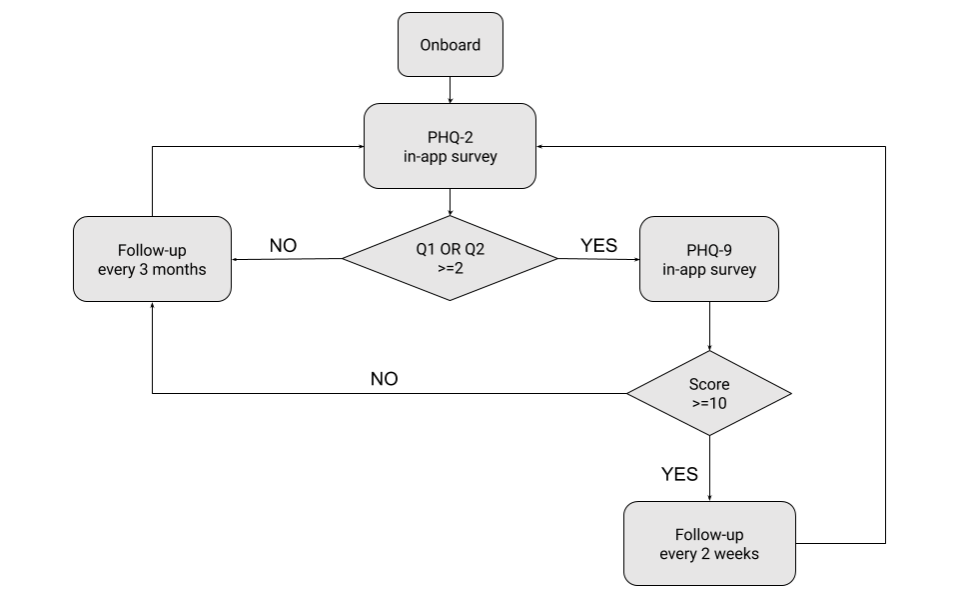
Ginger PHQ survey logic.

There are no strict guidelines on how often to re-administer the PHQ-2 and PHQ-9; it can be re-administered as needed with a standard recommendation for monitoring and adjusting treatment every 4-6 weeks for users seeking treatment for depression [29]. Similarly, according to the US Preventive Services Task Force (USPSTF), there is little evidence regarding the optimal and interval for screening for depression. The USPSTF recommends a pragmatic approach of screening all adults who have not been screened previously and “using clinical judgment in consideration of risk factors, comorbid conditions, and life events to determine if additional screening of high-risk patients is warranted” [30,31]. Based on this guidance, Ginger administers the survey every 2 weeks for users with PHQ-9 scores ≥10 and every 3 months for users with PHQ-9 scores <10 in order to monitor symptom response and assess if additional care is warranted (Figure 3). Survey completion is not forced so as not to withhold support from members who require care.

In addition to clinical outcomes, Ginger collects and analyzes product data to understand a member’s progress and journey through the system, including access, engagement, and member satisfaction.

### Measures

#### Engagement

Engagement is characterized based on product user behavior data, including the number of coaching sessions and the number of clinical (therapy and psychiatry) appointments attended. A coaching session is defined as 25 messages exchanged. This threshold is based on an internal analysis of messaging patterns in addition to coaching team feedback on what constitutes a typical session. These measures provide insight into dropoff, adherence, and dosing, which are essential areas of study in this field [32,33].

#### Member Satisfaction

Member Satisfaction is measured using a 5-star survey. Ginger regularly prompts users to rate their experience via “star ratings.” For coaching, this is required after the first 25 messages (ie, one text-based coaching session), and then every two weeks following. For clinical sessions, members are prompted to provide a rating after every therapy and psychiatry appointment. In addition to being a universal tool for consumers to rate products and services online (eg, Yelp, Amazon, Google), star ratings have also been used to understand provider care [34,35]. Although there are limitations associated with these consumer ratings, they can provide valuable insights for further exploration. Given the potential biases in these responses, the Ginger system includes proactive outreach from the member support team anytime there is less than a 4-star rating in addition to a clinical QA program to understand the care a member is receiving.

#### Depression Symptoms

Depression symptoms were assessed using the 2-item Patient Health Questionnaire (PHQ-2). The PHQ-2 comprises the first two items of the PHQ-9, assessing the degree to which an individual has experienced depressed mood and anhedonia over the preceding two weeks. A description of this survey logic is provided above. For this analysis, a negative depression screen means a user’s response to each PHQ-2 question was less than 2 (ie, a response of “not at all” or “several days”). A positive depression screen means a user’s response to either PHQ-2 question was ≥2 (ie, a response of “more than half the days” or “nearly every day”). Thus, a positive screen in this system can be interpreted to an individual having “little interest or pleasure in doing things” and “feeling down, depressed, or hopeless” more than half the days in the last 2 weeks. Although the PHQ-2 is generally used as a screening survey, it has a relatively high sensitivity for major depressive disorder, and previous research has utilized it as a longitudinal outcome measure [36-38]. Additionally, it provides insight into the severity of two key symptoms of depression: depressed mood and anhedonia. For this analysis, we considered a follow-up window of 8-12 weeks to reflect coaching and clinical protocols and the period in which we expect coaching and psychotherapy to have a measurable impact [32,33].

### Data Management and Analysis

Data for this study were processed using Looker, business intelligence, and data analytics platform. Data were analyzed in Python and exported to spreadsheets for final analysis and review. We first assessed descriptive statistics for eligible users and those who completed follow-up. We then looked at the percentage of users demonstrating a change in PHQ-2 screen from baseline to follow-up for those who completed in-app surveys. We further evaluated these changes from baseline to follow-up using the McNemar test. The McNemar test compares dependent samples in terms of a dichotomous variable and is used for pretest/posttest designs or in time series data where the same sample is tested at least in two points in time [39]. We determined this to be the most appropriate test given the study design and nature of the outcome variable (comparing PHQ-2 screen outcomes at baseline and follow-up).

To evaluate outcomes by care modality (coaching only, therapy + psychiatry, hybrid) for users who screened positive at intake, we performed a chi-squared test to see if the proportion of individuals with reduced depression symptoms (change from a positive screen at intake to negative at follow-up) differed between these groups.

### Ethics Statement

This study represents a secondary analysis of pre-existing de-identified data. The study team does not have access to the participants or participant identifying information and does not intend to recontact participants. This study protocol was reviewed by Advarra IRB and determined to be exempt from IRB oversight as de-identified secondary data analysis is generally not regarded as human subjects research.

## Results

Table 1 reports descriptive characteristics of the sample who completed follow-up. Of 1662 individuals included in this analysis, 46.5% (772/1662) screened positive for depression at intake, and 53.6% (890/1662) screened negative according to the PHQ-2 questionnaire.

Gender and demographic data were missing for a large portion of the sample, as this has historically been optional information provided in employer eligibility files. For those users who did have data reported (N=678), 68.7% (466/678) were female, and 30.8% (209/678) were male. Of the individuals that reported age information, 11.5% (122/1064) were 18-24, 51.3% (546/1064) were 25-34, 22.7% (242/1064) were 35-44, 14.3% (152/1064) were 45-64, and 0.1% (2/1064) were 65 or older. When looking at the modality of care in which individuals were engaged, 65.5% (1089/1662) were only engaged in coaching, 27.6% (458/1662) were engaged in both coaching and clinical (therapy or psychiatry) services, 3.3% (54/1662) were only engaged in clinical services, and 3.7% (61/1662) used the app but did not engage in coaching or clinical services.

For those individuals engaged in coaching, the average number of sessions was 6 over the study period. For those individuals engaged in any clinical service (therapy or psychiatry), the average number of appointments was 6 over the period. The average number of therapy appointments for those engaged in therapy services was 6. The average number of psychiatry appointments for those engaged in psychiatry services was 3.

Table 2 shows the frequency and percentage of users by PHQ-2 result at intake and follow-up. Of the 1662 individuals who completed follow-up, 772 (46.5%) were considered a positive screen at intake, and 890 (53.6%) were considered a negative screen at intake. At the follow-up window, 477 (28.7%) of individuals screened positive, and 1185 (71.3%) screened negative. Using the McNemar test, we concluded that there was a statistically significant difference in the proportion of users with a positive PHQ-2 result at baseline and follow-up, *P*<.001.

**Table 1:** Characteristics of the study cohort.

Characteristic		Value
**Gender, n (%)**		
	Female	466 (28.04)
	Male	209 (12.58)
	Other	3 (0.18)
	No response	984 (59.21)
**Age, n (%)**		
	18-24 years old	122 (7.34)
	25-34 years old	546 (32.85)
	35-44 years old	242 (14.56)
	45-64 years old	152 (9.15)
	65 or older	2 (0.12)
	No response	598 (35.98)
**Care modality, n (%)**		
	Coaching only	1089 (65.52)
	Hybrid (coaching + clinical)	458 (27.56)
	Clinical only	54 (3.25)
	App only	61 (3.67)
**Engagement, mean (SD)**		
	Coaching sessions	6.14 (5.44)
	Clinical appointments	5.79 (3.12)
	Therapy appointments	5.54 (2.8)
	Psychiatry appointments	2.71 (1.7)
**Member satisfaction, mean (SD)**		
	Coach star rating	4.63 (0.6)
	Clinical star rating	4.74 (0.61)
**Patient Health Questionnaire intake screen, n (%)**		
	Positive	772 (46.45)
	Negative	890 (53.55)

**Table 2:** Patient Health Questionnaire-2 (PHQ-2) results at intake and follow-up (N=1662).

PHQ-2 screen result (pre)	Post			Count, n		X^2^ (*df*)		*P* value
	Positive, n (%)	Negative, n (%)						
Positive	312 (18.77)	460 (27.68)	1662		139.24 (1)		<.001	
Negative	165 (9.93)	725 (43.62)						

^a^McNemar chi-squared test.

Table 3 shows the frequency and percentage of users who experienced a change in their PHQ-2 result by care modality for the users who screened positive at intake (N=748). Using a chi-square test, we concluded that there was not a significant association between the proportion of individuals with a positive screen at intake who improved at follow-up and care modality (*P*=.77).

**Table 3:** Association between PHQ-2 improvements and care modality (N=748).

Variable (care modality)	Change, n (%)	No change, n (%)	Count, n	X^2^ (*df*)	*P* value^a^
Coaching only	281 (58.54)	199 (41.46)	480	1.1289 (2)	.770
Therapy only	16 (61.54)	10 (38.46)	26		
Hybrid	150 (61.98)	92 (38.02)	242		

^a^Chi-square test for independence.

## Discussion

### Principal Findings

Although there is growing evidence that digital and virtual mental health interventions show promise in improving clinical outcomes for individuals with depression, many studies have highlighted the need to study further the features of specific technologies and the populations that use them. This study adds to this literature by describing a specific virtual on-demand mental health system and providing some preliminary results on user characteristics, engagement, and depression symptoms to guide future research.

Descriptive statistics showed that a majority of individuals were only utilizing coaching services (65.52%), and a minority of individuals were only utilizing clinical services (3.25%). The average coach and clinician star rating was >4.5, which provides some signal that members receiving care are satisfied with the care they are receiving, although more research is required to understand satisfaction with specific aspects of the system.

A McNemar test showed that the proportion of individuals with a positive PHQ-2 screen significantly decreased at follow-up, thus providing preliminary evidence that the Ginger system has an impact on decreasing depression symptom severity. A chi-square test concluded that there was not a significant association between care modality and the proportion of individuals with a positive screen improving at follow-up. While further research is required, this suggests that text-based behavioral health coaching alone can be an effective modality for reducing depression symptoms in addition to traditional clinical services like therapy and psychiatry. Based on the operationalization of our outcome variable, it is important to note that this analysis only evaluated change in PHQ-2 screen results; thus, we are underestimating the proportion of individuals who improved because we are not capturing individuals who screened positive at follow-up according to the PHQ-2 but still experienced a full symptom response according to PHQ-9.

### Limitations

This analysis has various limitations based on historical product and clinical design of the system that generated these observational data. First, PHQ survey completion is not required for all users. Thus, the cohort we were able to study was limited, and these outcomes are not necessarily generalizable to the overall user base due to bias in the users who complete the survey. Efforts to improve the product design, user experience, and integrations with clinical workflows will also allow us to study a broader cohort in the future and make more generalizable conclusions.

Given the historical survey design of this system, clinical outcomes were operationalized as a binary variable using PHQ-2, which can be interpreted as a positive or negative depression screen or severity of anhedonia and depressed mood. Thus, we could only measure outcomes as a dichotomous variable and did not assess the extent of symptom response on a continuous scale. Although the PHQ-2 is generally intended to be a screening survey, it has a relatively high sensitivity for major depressive disorder, and there is precedent for using it as a longitudinal outcome [36-38]. Furthermore, it explicitly measures the severity of two key symptoms of depression: anhedonia and depressed mood.

Although we have some demographic data on users via employer eligibility files, missing data meant we were not able to stratify our analyses or control for specific demographic variables since users are not currently required to provide this information directly to Ginger. Future product updates will address this missing data issue and allow stratified analyses of outcomes by demographics (gender, age, socioeconomic status) to help us better understand how outcomes differ for specific populations.

Finally, because we lacked a control group, we are unable to understand the significance of these outcomes versus usual care or no care. Given ethical challenges, few prospective non-intervention studies following the natural course of untreated depression exist [37]. Prior research looking at wait-list control groups has suggested that 20-25 percent of untreated depression cases will remit within 3 months [40,41].

Collectively, these limitations point to many directions for future research. By using this dataset, follow-up studies could examine which aspects of this system are associated with changes in clinical outcomes, eg, different types of coaching and therapy, different thresholds of engagement, frequency of coach and clinical interaction, and different types of in-app content. New measurements and survey tools such as productivity, quality of life, and functional outcomes will also help evaluate impact in a broader cohort of users where the PHQ survey is not the most appropriate measure of progress, eg, those who screen negatively for depression. Finally, randomized controlled studies could also build upon this research and further test hypotheses on the efficacy of specific interventions in this system.

### Conclusion

There is growing evidence that telehealth and other digital interventions can be useful in reducing symptoms of depression and other mental health conditions. This study adds to the literature by describing a specific on-demand mental health system and investigating utilization patterns and impact. The results of this exploratory study show a significant decrease in the proportion of users experiencing depressed mood and anhedonia at follow-up. Limitations with this study design mean that these results are not generalizable to the entire user base nor attributable to a specific intervention. Future studies can address these limitations and provide additional insight into which features of the system are most associated with outcomes in different populations.

## Abbreviations

CMS: Centers for Medicare & Medicaid Services
MBC: measurement-based care
PHQ: Patient Health Questionnaire

## References

[1] National Institute of Mental Health.

[2] Brody D, Pratt L, Hughes J National Center for Health Statistics.

[3] Blue Cross Blue Shield (BCBS).

[4] Chow W, Doane M, Sheehan J, Alphs L, Le H (2019). Economic Burden Among Patients With Major Depressive Disorder: An Analysis of Healthcare Resource Use, Work Productivity, and Direct and Indirect Costs by Depression Severity. American Journal of Managed Care.

[5] Cunningham PJ (2009). Beyond parity: primary care physicians' perspectives on access to mental health care. Health Aff (Millwood).

[6] Kaiser Family Foundation.

[7] Thomas KC, Ellis AR, Konrad TR, Holzer CE, Morrissey JP (2009). County-level estimates of mental health professional shortage in the United States. Psychiatr Serv.

[8] Waitzfelder B, Stewart C, Coleman K, Rossom R, Ahmedani B, Beck A, Zeber J, Daida Y, Trinacty C, Hubley S, Simon G (2018). Treatment Initiation for New Episodes of Depression in Primary Care Settings. J Gen Intern Med.

[9] Lin P, Campbell DG, Chaney EF, Liu C, Heagerty P, Felker BL, Hedrick SC (2005). The influence of patient preference on depression treatment in primary care. Ann Behav Med.

[10] Raue P, Schulberg H, Heo M, Klimstra S, Bruce M (2009). Patients' depression treatment preferences and initiation, adherence, and outcome: a randomized primary care study. Psychiatr Serv.

[11] Gilbody S, Bower Peter, Fletcher Janine, Richards David, Sutton Alex J (2006). Collaborative care for depression: a cumulative meta-analysis and review of longer-term outcomes. Arch Intern Med.

[12] Overbeck G, Davidsen AS, Kousgaard MB (2016). Enablers and barriers to implementing collaborative care for anxiety and depression: a systematic qualitative review. Implement Sci.

[13] (2018). Kaiser Family Foundation.

[14] Godleski L, Darkins A, Peters J (2012). Outcomes of 98,609 U.S. Department of Veterans Affairs patients enrolled in telemental health services, 2006-2010. Psychiatr Serv.

[15] Coughtrey AE, Pistrang N (2016). The effectiveness of telephone-delivered psychological therapies for depression and anxiety: A systematic review. J Telemed Telecare.

[16] Firth J, Torous J, Nicholas J, Carney R, Pratap A, Rosenbaum S, Sarris J (2017). The efficacy of smartphone-based mental health interventions for depressive symptoms: a meta-analysis of randomized controlled trials. World Psychiatry.

[17] Safavi K, Cohen A (2019). Health Affairs.

[18] Henson P, David G, Albright K, Torous J (2019). Deriving a practical framework for the evaluation of health apps. The Lancet Digital Health.

[19] Bowen DJ, Kreuter M, Spring B, Cofta-Woerpel L, Linnan L, Weiner D, Bakken S, Kaplan CP, Squiers L, Fabrizio C, Fernandez M (2009). How we design feasibility studies. Am J Prev Med.

[20] Peters DH, Adam T, Alonge O, Agyepong IA, Tran N (2014). Implementation research: what it is and how to do it. Br J Sports Med.

[21] Marcelle ET, Nolting L, Hinshaw SP, Aguilera A (2019). Effectiveness of a Multimodal Digital Psychotherapy Platform for Adult Depression: A Naturalistic Feasibility Study. JMIR Mhealth Uhealth.

[22] Owen JE, Jaworski BK, Kuhn E, Makin-Byrd KN, Ramsey KM, Hoffman JE (2015). mHealth in the Wild: Using Novel Data to Examine the Reach, Use, and Impact of PTSD Coach. JMIR Ment Health.

[23] Nulty DD (2008). The adequacy of response rates to online and paper surveys: what can be done?. Assessment & Evaluation in Higher Education.

[24] Pratap A, Neto EC, Snyder P, Stepnowsky C, Elhadad N, Grant D, Mohebbi MH, Mooney S, Suver C, Wilbanks J, Mangravite L, Heagerty PJ, Areán Pat, Omberg L (2020). Indicators of retention in remote digital health studies: a cross-study evaluation of 100,000 participants. NPJ Digit Med.

[25] National Board for Health and Wellness Coaching.

[26] Institute of Coaching.

[27] Lewis CC, Boyd M, Puspitasari A, Navarro E, Howard J, Kassab H, Hoffman M, Scott K, Lyon A, Douglas S, Simon G, Kroenke K (2019). Implementing Measurement-Based Care in Behavioral Health: A Review. JAMA Psychiatry.

[28] American Psychological Association.

[29] Simon GE, Rutter CM, Peterson D, Oliver M, Whiteside U, Operskalski B, Ludman EJ (2013). Does response on the PHQ-9 Depression Questionnaire predict subsequent suicide attempt or suicide death?. Psychiatr Serv.

[30] Maurer DM, Raymond TJ, Davis BN (2018). Depression: Screening and Diagnosis. Am Fam Physician.

[31] United States Preventive Services Taskforce.

[32] Nieuwsma JA, Trivedi RB, McDuffie J, Kronish I, Benjamin D, Williams JW (2012). Brief Psychotherapy for Depression: A Systematic Review and Meta-Analysis. Int J Psychiatry Med.

[33] Maeng D, Cornell AE, Nasra GS (2019). Utilization of an Employee Behavioral Health Program and Its Effects on Outcomes for Depression and Anxiety Disorders. Journal of Occupational and Environmental Medicine.

[34] Ranard BL, Werner RM, Antanavicius T, Schwartz HA, Smith RJ, Meisel ZF, Asch DA, Ungar LH, Merchant RM (2016). Yelp Reviews Of Hospital Care Can Supplement And Inform Traditional Surveys Of The Patient Experience Of Care. Health Aff (Millwood).

[35] Donnally CJ, Roth ES, Li DJ, Maguire JA, McCormick JR, Barker GP, Rivera S, Lebwohl NH (2018). Analysis of Internet Review Site Comments for Spine Surgeons. SPINE.

[36] Arroll B, Goodyear-Smith F, Crengle S, Gunn J, Kerse N, Fishman T, Falloon K, Hatcher S (2010). Validation of PHQ-2 and PHQ-9 to screen for major depression in the primary care population. Ann Fam Med.

[37] Fleishman J, Zuvekas S, Pincus H (2014). Agency for Healthcare Research and Quality.

[38] Löwe Bernd, Kroenke K, Gräfe Kerstin (2005). Detecting and monitoring depression with a two-item questionnaire (PHQ-2). J Psychosom Res.

[39] Fisher MJ, Marshall AP, Mitchell M (2011). Testing differences in proportions. Aust Crit Care.

[40] Casacalenda N, Perry JC, Looper K (2002). Remission in major depressive disorder: a comparison of pharmacotherapy, psychotherapy, and control conditions. Am J Psychiatry.

[41] Whiteford HA, Harris MG, McKeon G, Baxter A, Pennell C, Barendregt JJ, Wang J (2012). Estimating remission from untreated major depression: a systematic review and meta-analysis. Psychol. Med.

